# Policewomen

**Published:** 1912-07

**Authors:** 


### BUFFALO MEDICAL JOURNAL

**A Monthly Review of Medicine and Surgery**

*EDITOR*

A. L. BENEDICT.

All communications, whether of a literary or business nature, books for review and exchanges, should be addressed to the editor, 354 Franklin Street, Buffalo, N. Y Please make personal or telephonic calls before 1 p. m

The Buffalo Medical Journal will publish regularly, full transactions of any professional organization in western New York at cost. Personal notices, brief reports of society proceedings, and the like, are always welcome and will be published without charge.

Subscriptions may commence at any time. $2.00 per year in advance,

Vol. LXVII. JULY, 1912 No. 12

*The dollar is measured by its purchasing power. Do not let it buy of you anything that you can not afford to lose.*

### Policewomen

The advocacy of policewomen originated with Mrs. Julia Goldzier, of Bayonne, N. J., and we understand that such a force has actually been put into service in Los Angeles and Indianapolis and some other western towns. Mrs. Goldzier's idea is, at the beginning, to confine this service to public play grounds, including parks, the vicinity of schools at the time the children are congregating or dispersing to their homes. After a thorough practical test has been made, other much wider fields for the service may develop, not only in the geographic sense but in regard to the extension of the duties of the policewomen to cover more serious misdemeanors than children's quarrels and petty acts of dishonesty.

The gist of Mrs. Goldzier's argument is that children are as valuable to the state as property, quite as much in need of protection as the latter, that women are required to guarantee this protection. The first two points will readily be admitted; the only difference of opinion being with regard to whether children require special oversight beyond that provided by parents, teachers and other existing agencies, and whether the present male police force can adequately provide such oversight. With the inevitable tendency to concentration of population, carrying with it a greater and greater restriction of private play grounds, it is obvious that more and more of the necessary outdoor life of the child and, more particularly the child of the tenement district must be spent in the public streets, parks and play grounds. That the child needs guarding against physical risk, against moral contamination, and against the insidious development of criminal tendencies, even against kidnapping, seems to us to require no further argument than observation and study of reports of crime. As to whether the parent, the teacher and other existing guardians of the child can provide the necessary supervision throughout the entire day, we need only ask the reader of more than average circumstances, whether his own child is protected for the entire period of risk and then reflect on what the conditions must be for the child of the already large and constantly increasing class in which both parents must be wage earners, or in which the domestic duties of the mother are so heavy as to preclude much attention to children after their early infancy.

It may, of course, be argued that the existing male police force is all that the state should provide to prevent law breaking by persons of any age and to breed a respect for law and for its underlying moral principles. Here again, actual observed facts are more eloquent than theory. The fact is that children in the most impressionable period of their development do not come within the jurisdiction of the policeman, to any adequate degree and, if we pause to consider the reason, we must acknowledge that the present police force exists largely as a preliminary punitive arm of the law, to deal with actual offenses rather than tendencies toward crime and that, without losing prestige and efficiency, it cannot, even allowing for necessary numeric increase, deal with the petty quarrels of children or supplement the moral training of the school and home, except incidentally and occasionally.

Mrs. Goldzier anticipates a good many arguments of those prejudiced against the extension of woman's work. The arguments on both sides are too familiar in these days to need repetition.

